# A GelMA-PEGDA-nHA Composite Hydrogel for Bone Tissue Engineering

**DOI:** 10.3390/ma13173735

**Published:** 2020-08-24

**Authors:** Yihu Wang, Xiaofeng Cao, Ming Ma, Weipeng Lu, Bing Zhang, Yanchuan Guo

**Affiliations:** 1 Key Laboratory of Photochemical Conversion and Optoelectronic Materials, Technical Institute of Physics and Chemistry, Chinese Academy of Sciences, Beijing 100190, China; wyh8632@mail.ipc.ac.cn (Y.W.); xfcao@mail.ipc.ac.cn (X.C.); maming@mail.ipc.ac.cn (M.M.); luweipeng@mail.ipc.ac.cn (W.L.); 2 Hangzhou Branch of Technical Institute of Physics and Chemistry, Chinese Academy of Sciences, Hangzhou 310018, China

**Keywords:** hydrogel, GelMA, nHA, photo-crosslinking

## Abstract

A new gelatin methacrylamine (GelMA)-poly (ethylene glycol) diacrylate (PEGDA)-nano hydroxyapatite (nHA) composite hydrogel scaffold was developed using UV photo-crosslinking technology. The Ca^2+^ from nHA can form a [HO]Ca^2+^ [OH] bridging structure with the hydroxyl group in GelMA, thereby enhancing the stability. Compared with GelMA-PEGDA hydrogel, the addition of nHA can control the mechanical properties of the composite hydrogel and reduce the degradation rate. In vitro cell culture showed that osteoblast can adhere and proliferate on the surface of the hydrogel, indicating that the GelMA-PEGDA-nHA hydrogel had good cell viability and biocompatibility. Furthermore, GelMA-PEGDA-nHA has excellent injectability and rapid prototyping properties and is a promising 3D printed bone repair scaffold material.

## 1. Introduction

Bone repair is a dynamic process in which osteoprogenitor cells are recruited to the bone defect site by a combination of various cytokines and growth factors, which are then guided to differentiate into osteoblasts [1,2]. However, for patients with severely injured bone defects, osteoporosis or congenital skeletal deformities, the process of bone self-healing is slow and limited [3,4]. Therefore, manual intervention is necessary to increase bone mass, such as bone transplantation, bone cement or medication. Due to the risk of disease transmission, infection and rejection, autologous bone graft and allogeneic bone transplantation are currently not widely used in clinical practice [5].

Bone tissue engineering is proposed on this basis and is a new method to promote bone repair and regeneration [6]. The scaffold material used provides structural support, promotes cell adhesion, proliferation, and creates favorable conditions for differentiation, thereby achieving bone mass increase and functional recovery at the bone defect site [7,8]. Common bone tissue scaffold materials can be divided into artificial synthetic materials, natural derived materials, and composite scaffold materials. Artificial synthetic materials include inorganic materials such as hydroxyapatite [9,10], tricalcium phosphate [11], and organic materials such as polylactic acid [12] and polyethylene glycol [13]. Naturally derived materials include collagen [14], chitosan [15], coral [16], cuttlefish bone [17], etc. The composite scaffold materials are composed of a variety of synthetic materials and natural derived materials.

Hydroxyapatite (HA) is the main inorganic component of human bones, and is a widely used inorganic material in bone tissue engineering [9,10]. As an implant, HA has good stability, biocompatibility, and degradability. HA can not only promote the adhesion, proliferation of osteoblasts, and extracellular matrix secretion, but also form chemical bonds with the body’s own bones [18,19,20]. Therefore, it has the effect of repairing bone defects.

Hydrogel is a hydrophilic polymer scaffold with many excellent properties, controllable mechanical properties and can provide a nutrient environment for endogenous cell growth, and can simulate the natural extracellular matrix of bones [21,22]. Hydrogels can be customized to obtain the desired geometry for implantation or injection. Many synthetic polymers and natural derivative materials can be used to synthesize hydrogels [21,23]. By modifying the method and degree of cross-linking, the degradation rate, porosity, and mechanical properties can be easily controlled [24]. The hydrogel prepared by combining natural polymer, synthetic polymer, and HA has achieved good results in bone repair experiments [25,26]. Radhakrishnan [27] prepared a hydrogel composed of alginate, chitosan, polyethylene glycol, and nHA, which showed good immunocompatibility and biocompatibility. Fu [28] developed a new three-component bionic hydrogel composite material consisting of triblock PEG-PCL-PEG copolymer, collagen, and nHA. The New Zealand White Rabbit skull model test proved that this composite hydrogel can guide bone regeneration, which has good biocompatibility and better performance than the self-healing process [28].

In this work, combined with the excellent physicochemical properties and biocompatibility of GelMA-PEGDA hydrogel [29], GelMA-PEGDA-nHA ternary composite hydrogel was prepared by doping nHA into prepolymer. nHA is expected to increase the mechanical strength of the hydrogel, and the photo-crosslinking properties of GelMA-PEGDA give the material advantages in processing and injectability. The physicochemical properties and biocompatibility of GelMA-PEGDA-nHA hydrogel were systematically studied.

## 2. Materials and Methods

### 2.1. Materials

GelMA (degree of modification 76%) was self-made [29], poly (ethylene glycol) diacrylate (PEGDA), 2-hydroxy-1-(4-(hydroxyethoxy) phenyl)-2-methyl-1-propanone (Irgacure 2959), and deuterium oxide were purchased from Sigma-Aldrich (St. Louis, MO, USA). nHA (<100 nm particle size) was purchased from Aladdin (Shanghai, China). Collagenase type I were purchased from Solarbio (Beijing, China). MC3T3-E1 (mouse osteoblast cell line, six passages), fetal bovine serum, Alpha Modification Eagle Medium (α-MEM), and PBS buffer (pH 7.4) were purchased from Union Hospital (Beijing, China). The live/dead assay kit was purchased from ABcam (Britain, UK). All other reagents and solvents were of reagent grade.

### 2.2. Preparation of GelMA-PEGDA-nHA Composite Hydrogel

The proportional relationship between the different components is shown in Table 1. Irgacure 2959 was added to deionized water, thermostated to 60 °C in a constant temperature water bath, and then ultrasonically dispersed to completely dissolve. Different amounts of nHA were added to deionized water and ultrasonically dispersed. GelMA was added to cold deionized water, swollen for 1 h, and then placed in a constant temperature water bath at 60 °C. After 1 h, the solution was ultrasonically dispersed until completely dissolved. PEGDA was added to the dissolved aqueous solution of GelMA and ultrasonically dispersed until completely dissolved. The solutions were mixed, protected from light, and ultrasonicated until completely dissolved and defoamed. The reaction solution was quickly poured into a mold (10 mm in diameter, 8 mm in height) and placed on a constant temperature reaction table at 60 °C. Under the irradiation of a UV lamp, the crosslinking reaction was carried out for 10 min. The hydrogel sample was taken out of the mold and quickly rinsed twice with deionized water to remove the unreacted material.

### 2.3. Scanning Electron Microscope Analysis

The prepared GelMA-PEGDA-nHA hydrogel was placed in a PBS solution, equilibrated at 37 °C for 24 h, and the surface moisture was taken out and frozen at −80 °C for 24 h, followed by freeze-drying. The obtained dried sample was cut into thin pieces and sprayed with gold to perform a scanning electron microscope test. The pore size and wall thickness were counted using the Image J software, (NIH, Bethesda, MD, USA) three images were selected for each sample, and each image measures 30 holes.

### 2.4. FT-IR Analysis

The freeze-dried GelMA-PEGDA-nHA hydrogel was subjected to ATR-FT-IR, and the scanning range was 400–4000 cm^−1^, the resolution was 4 cm^−1^, the number of scans was 32, and the infrared spectrum was measured. FT-IR is used to study the chemical bond between HA and GelMA-PEGDA.

### 2.5. Swelling Ratio

The hydrogel was immersed in PBS for 24 h at 37 °C to fully swell, and its swelling weight *W_s_* was measured. Then, the hydrogel was lyophilized to obtain dry weight *W_d_*. The swelling degree was calculated as the following equation [30]:

(1) $$Swelling\;Ratio=\frac{W_{s}-W_{d}}{W_{d}}$$

### 2.6. Compressive Mechanical Properties

The compression performance of GelMA-PEGDA-nHA hydrogel was measured using a universal material testing machine (Instron 5960) (Boston, MA, USA). The compression speed was 0.1 mm min^−1^. Before the test, the sample was cut into a cylinder of 10 mm in diameter and 5 mm in height and swelled in a PBS buffer for 24 h, six samples were tested for each hydrogel. The hydrogel sample was placed on the test platform and the test ends when the sample was broken.

### 2.7. In Vitro Degradation

The GelMA-PEGDA-nHA hydrogel sample was freeze-dried, weighed, recorded as *W*_0_, and then the sample was swelled in a PBS solution, and the swollen sample was placed in a 15 mL centrifuge tube, and each tube was added with 5 mL of PBS solution (containing 2 UmL^−1^ type I collagenase), then the tube was placed in a 37 °C constant temperature water bath shaker, and the enzyme solution was changed every two days to maintain the enzyme activity. At 1, 3, 5, 7, 14, 21, 28, 42, 56 days, the samples were removed and rinsed twice with deionized water, then lyophilized and weighed and recorded as *W_t_*.

(2) $$Degradation\;Ratio=\frac{W_{0}-W_{t}}{W_{0}}$$

### 2.8. D Cell Culturing

The MC3T3-E1 cell line was added to the ⍺-MEM medium (10% FBS) and cultured in a constant temperature and humidity of 5% carbon dioxide incubator at 37 °C. The medium was changed every other day. The cells were digested with trypsin, collected, and the cell density was calculated using a cell counter.

The GelMA-PEGDA-nHA prepolymerization solution was sterilized with a 0.22 μm filter membrane, and then 150 μm prepolymerization solution was added to each well of a 24-well plate and irradiated under 365 nm ultraviolet light for 10 min to form a hydrogel. The hydrogel samples were washed twice with the PBS and α-MEM medium, respectively, and then 2 × 10^4^ MC3T3-E1 cells were added to each well, and live/dead cells were stained in one, three, and seven days, respectively and the state of the cells was observed by the inverted fluorescence microscope (DMI-6000B Leica) (Wetzlar, Germany). Three samples per group and three fields of each sample were used to calculate the cells viability.

### 2.9. Statistical Analysis

All experimental results were expressed as the mean ± standard deviation. GraphPad Prism version 7 (GraphPad Software, San Diego, CA, USA) was used for statistical analysis. The differences between group means were analyzed by the Student’s t-test and the significance level was set to *p* < 0.05. Cell viability was analyzed by the ImageJ software.

## 3. Results and Discussion

### 3.1. Preparation of GelMA-PEGDA-nHA Composite Hydrogel

**Figure 1:**
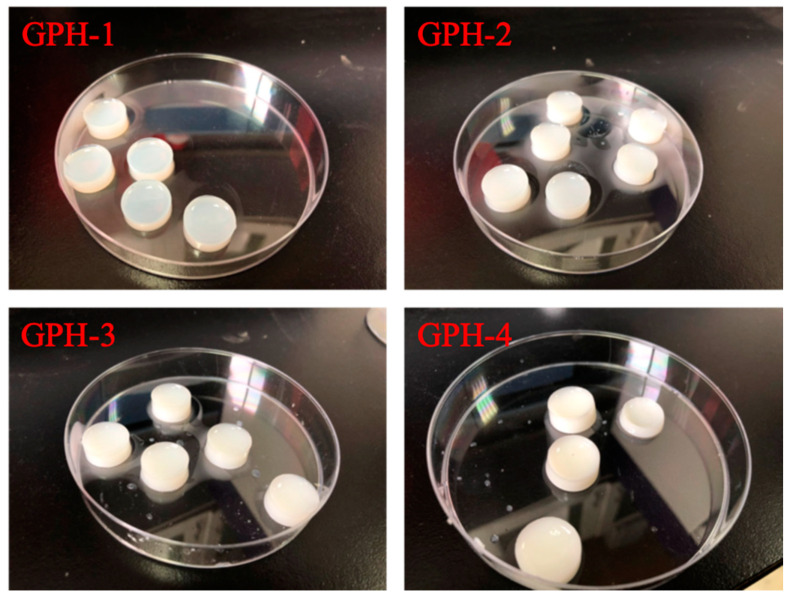
Gelatin methacrylamine-poly (ethylene glycol) diacrylate-nano hydroxyapatite (GelMA-PEGDA-nHA) composite hydrogel with different nHA contents.

Four different GelMA-PEGDA-nHA composite hydrogels were prepared according to the composition ratios in Table 1, as shown in Figure 1. As the content of nHA increases, the color of the composite hydrogel gradually changes from transparent to milky white.

### 3.2. Morphology of GelMA-PEGDA-nHA Hydrogel

**Figure 2:**
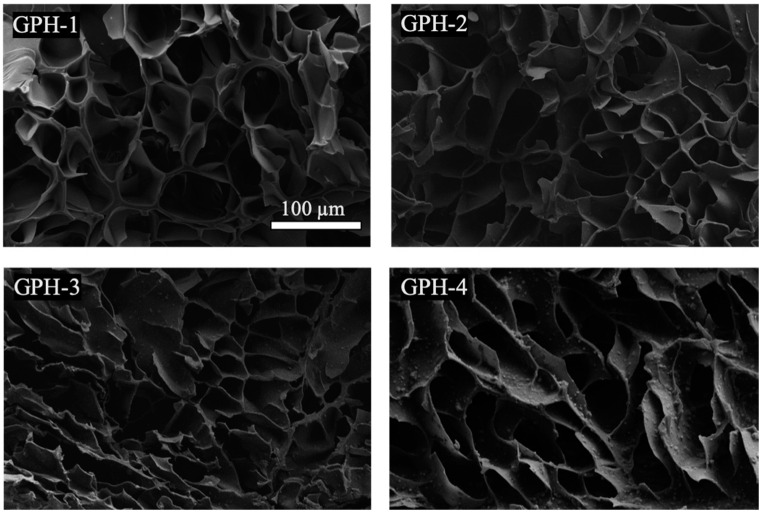
SEM image of GelMA-PEGDA-nHA composite hydrogel with different nHA contents.

**Figure 3:**
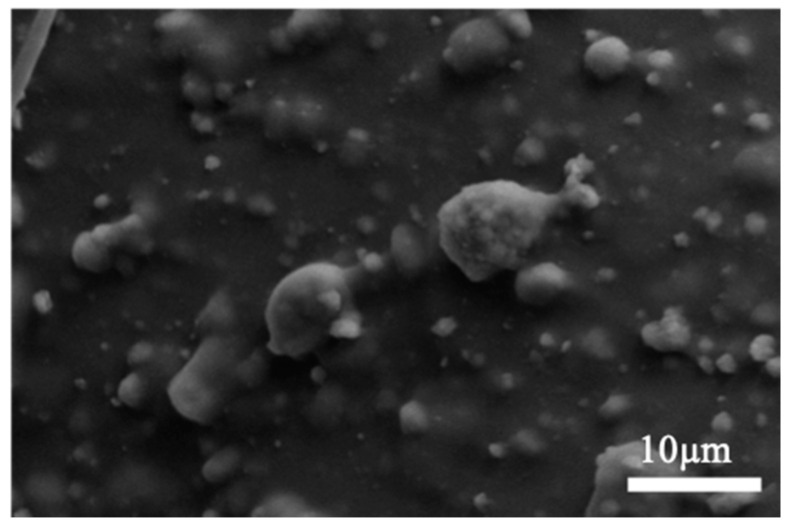
Partial enlargement of GelMA-PEGDA-nHA-4 sample.

A sample of the prepared GelMA-PEGDA-nHA composite hydrogel was subjected to lyophilization, and a photograph of the section was observed by SEM. As can be seen from Figure 2, GelMA and PEGDA were well combined and intertwined to form a network structure without a distinct interface. It can be observed that with the increase of the amount of nHA added, the number of small particles that were wrapped with GelMA-PEGDA on the surface of the pore wall of the lyophilized sample gradually increase, and the partial enlarged image is shown in Figure 3. The Ca/P ratio of the small particles in four samples were analyzed by EDS to almost be 1.6, which can be determined as HA.

**Figure 4:**
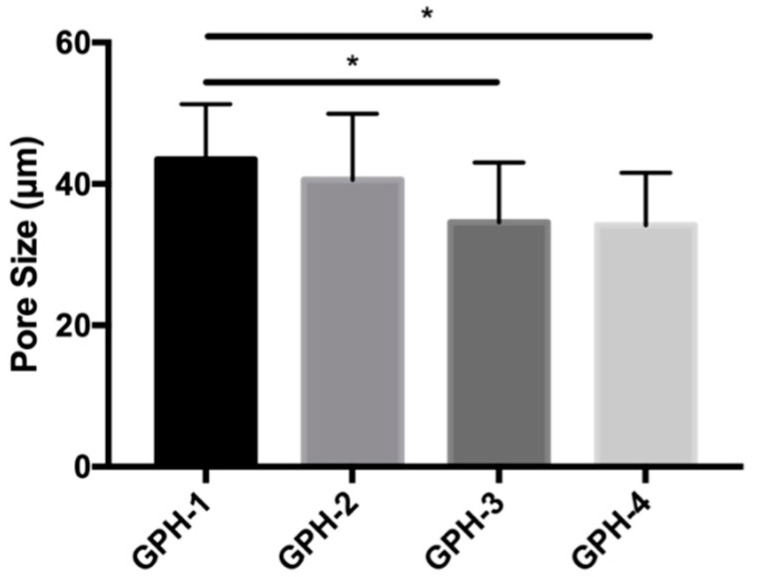
Pore size statistics of GelMA-PEGDA-nHA composite hydrogels with different nHA contents. (* *p* < 0.05).

The pore size change of the GelMA-PEGDA-nHA composite hydrogel was counted by the Image J software, as shown in Figure 4. The pore diameters of GPH-1, GPH-2, GPH-3, and GPH-4 were 43.45 ± 7.45, 40.59 ± 9.36, 34.59 ± 8.45, and 34.17 ± 7.43 μm, respectively. With the increase of nHA content, the pore size of the composite hydrogel showed a downward trend, and the composite hydrogel with 2% and 5% of nHA was significantly decreased compared with the composite hydrogel with 0.1% of nHA (*p* < 0.05). The thickness of the pore wall of the statistical composite hydrogel did not change significantly with the increase of the nHA content.

### 3.3. FT-IR Characterization

**Figure 5:**
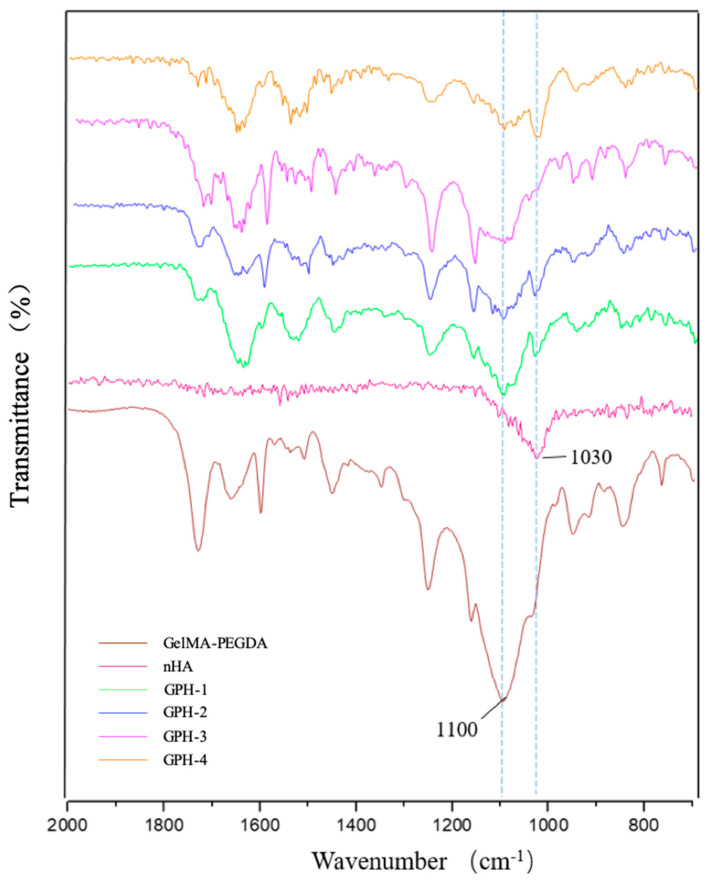
Infrared spectrum of nHA, GelMA-PEGDA, and GelMA-PEGDA-nHA.

Infrared spectroscopy was performed on nHA, GelMA-PEGDA hydrogel, and GelMA-PEGDA-nHA hydrogel, respectively, as shown in Figure 5. In the infrared spectrum of nHA, the stretching vibration peak of the phosphoric acid group P-O in hydroxyapatite was at 1030 cm^−1^. With the increase of nHA content in the ternary composite hydrogel, the characteristic absorption peak at 1030 cm^−1^ gradually increased. At the same time, we observed that the C-O stretching vibration peak 1100 cm^−1^ of the strong hydroxyl group in the GelMA-PEGDA hydrogel gradually weakened or disappeared in the GelMA-PEGDA-nHA ternary composite hydrogel as the content of nHA increased. This phenomenon may be due to the interaction of more and more Ca^2+^ ions with the -OH in the hydrogel as the nHA content increases. Based on these results, it can be inferred that nHA interacts with GelMA-PEGDA through hydrogen bonding to form a bridge structure of [HO]–Ca^2+^–[OH] [31], which enhances the mechanical properties and stability of the material to some extent.

### 3.4. Swelling Ratio

**Figure 6:**
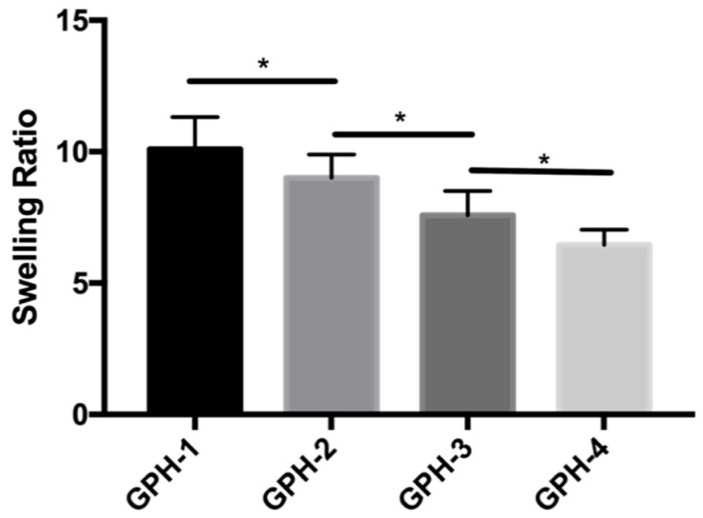
Swelling ratio of GelMA-PEGDA-nHA composite hydrogel with different nHA contents. (* *p* < 0.05).

As shown in Figure 6, the swelling ratio of GPH-1 was 10.09 ± 1.23, GPH-2 was 9.01 ± 0.88, GPH-3 was 7.58 ± 0.92, and GPH-4 was 6.45 ± 0.58. According to our previous research [29], the swelling rate of GP hydrogel with the same composition without nHA is 9.11 ± 0.14, compared with GPH-1, there is no significant difference. With the increase of the content of nHA in the composite hydrogel, the swelling ratio decreased significantly (*p* < 0.05). On the one hand, the added nHA itself has no swelling effect, resulting in a decrease in the overall swelling ratio. On the other hand, the addition of nHA causes the porosity of the hydrogel to decrease, and the water retention capacity to decrease, resulting in a decrease in the swelling rate. Moreover, the increase in nHA content enhances the effect between Ca^2+^ and GelMA-PEGDA hydrogel, thereby increasing the rigidity of the hydrogel, which makes the hydrogel difficult to swell and deforms to absorb more water.

### 3.5. Mechanical Properties

**Figure 7:**
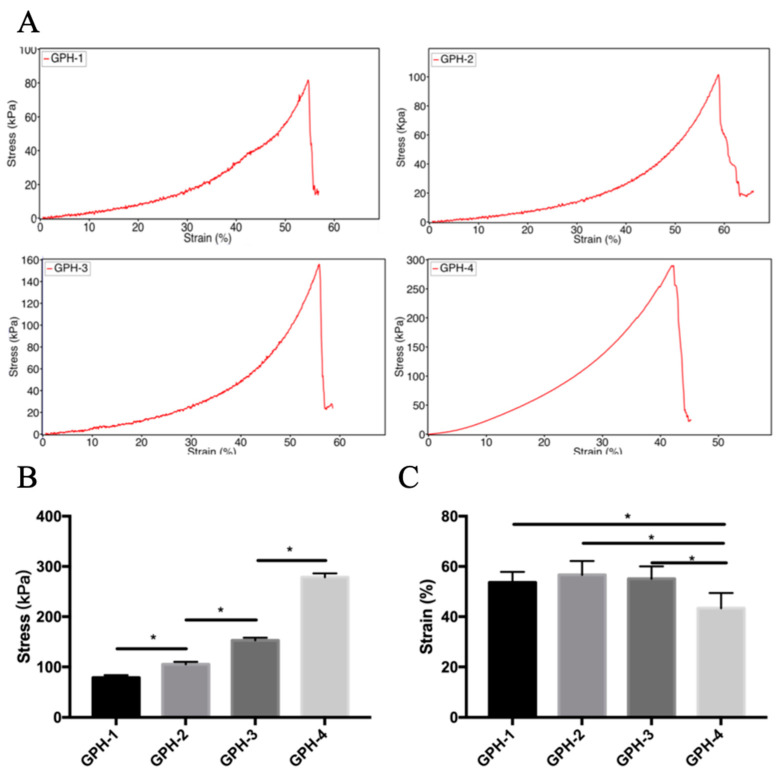
(**A**) Compressive stress-strain curve of GelMA-PEGDA-nHA composite hydrogel; (**B**) maximum compressive stress statistics; (**C**) maximum compressive strain statistics. (* *p* < 0.05).

The mechanical properties of GelMA-PEGDA-nHA composite hydrogels were investigated as a function of the amount of nHA added, as shown in Figure 7. In our previous study [29], the maximum compressive stress of GelMA-PEGDA hydrogel with the same composition is 70.60 ± 4.73 kPa. The maximum compressive stress increased significantly with the increase of nHA content in the composite hydrogel (*p* < 0.05). When the amount of nHA added is 0.1%, the maximum compressive stress is 79.25 ± 4.93 kPa, and when the amount of nHA added is 5%, the maximum compressive stress is 278.62 ± 7.49 kPa, which is more than three times. According to the previous SEM morphology analysis, the wall thickness of the composite hydrogel did not change significantly with the addition of nHA. The increase of compressive stress was mainly due to the increase of the rigidity of the hydrogel wall due to the addition of nHA. With the increase of rigidity, the compressive strain showed a downward trend with the increase of nHA content, and the strain of GPH-4 was significantly lower than that of the other three nHA content composite hydrogel samples (P<0.05). The mechanical properties also correspond to the results of the swelling rate. The samples with higher swelling rate have a greater compressive strain, but the compressive stress is lower. On the contrary, the samples with low swelling rate have a higher compressive stress, and the compressive strain is smaller. The uniform dispersion of nHA in the hydrogel network has a large effect on the mechanical properties. The compressive strength of the composite hydrogel is positively related to the content of nHA, so by changing the content of nHA, the mechanical properties of the composite hydrogel can be adjusted to meet the needs of different applications.

### 3.6. In Vitro Degradation Performance

**Figure 8:**
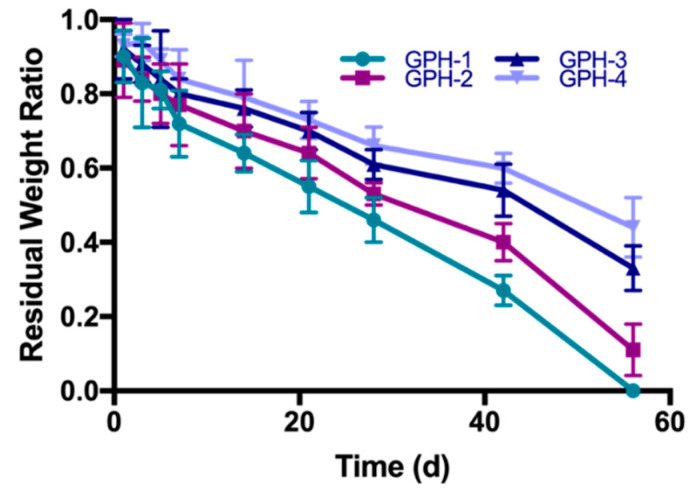
GelMA-PEGDA-nHA composite hydrogel degraded in type I collagenase solution.

The degradation of GelMA-PEGDA-nHA composite hydrogel in type I collagenase solution is shown in Figure 8. In our previous research [29], the residual rate of GelMA-PEGDA with the same composition on day 21 is 43.7% ± 3.2%. The residual rates of GPH-1, GPH-2, GPH-3, and GPH-4 on day 21 were 55.1% ± 7.2%, 64.3% ± 7.0%, 70.0% ± 5.1%, and 73.5% ± 5.6%, respectively. At 21 days, the residual rate of all GPH hydrogels containing nHA was significantly higher than that of GP hydrogels without nHA. GPH-1 completely degraded within eight weeks, while GPH-4 had an excess mass of more than 50% after eight weeks of in vitro degradation. In the case of the same GelMA and PEGDA content, increasing the content of nHA can significantly prolong the degradation time of the composite hydrogel. The degradation rate of GelMA-PEGDA-nHA was slower than the GelMA-PEGDA hydrogel previously reported. On the one hand, the degradation rate of nHA was slower than that of GelMA and PEGDA. On the other hand, Ca^2+^ in nHA can coordinate with the amide bond of gelatin [32], thereby increasing the stability of the composite hydrogel and prolonging the degradation time. The ratio of GelMA, PEGDA, and nHA in the composite hydrogel can be changed to control the degradation rate to meet the needs of different applications.

### 3.7. Biocompatibility

**Figure 9:**
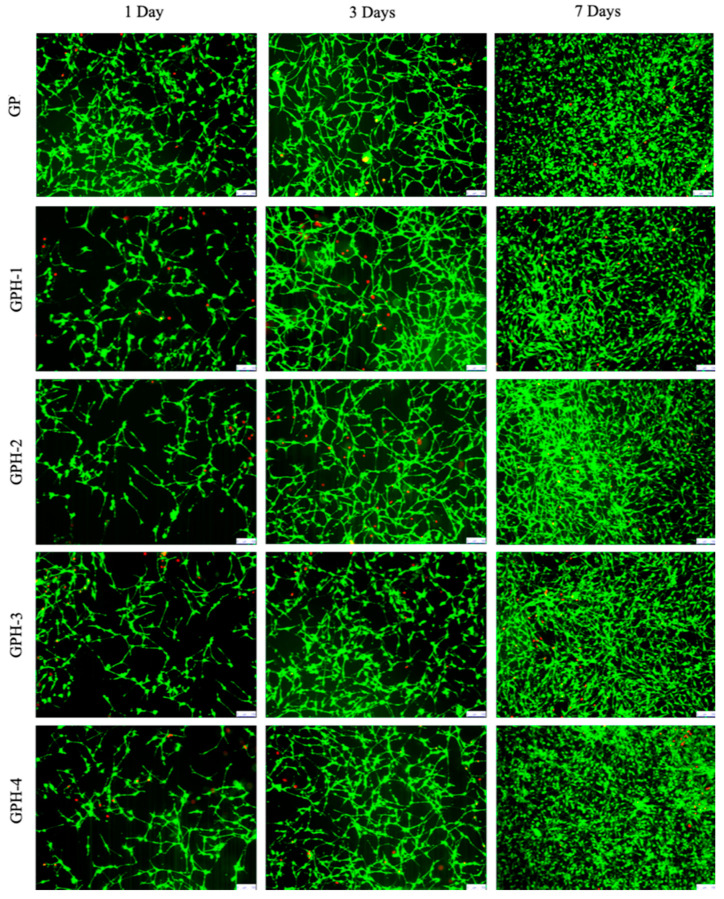
Live/dead staining of MC3T3-E1 on the surface of composite hydrogel (bar length 100 μm).

MC3T3-E1 cells were seeded on the surface of different nHA-containing GelMA-PEGDA-nHA composite hydrogels, and cells were stained by the Live-Dead Cell Staining Kit at 1, 3, and 7 d, respectively. The growth of the cells was observed, as shown in Figure 9.

**Figure 10:**
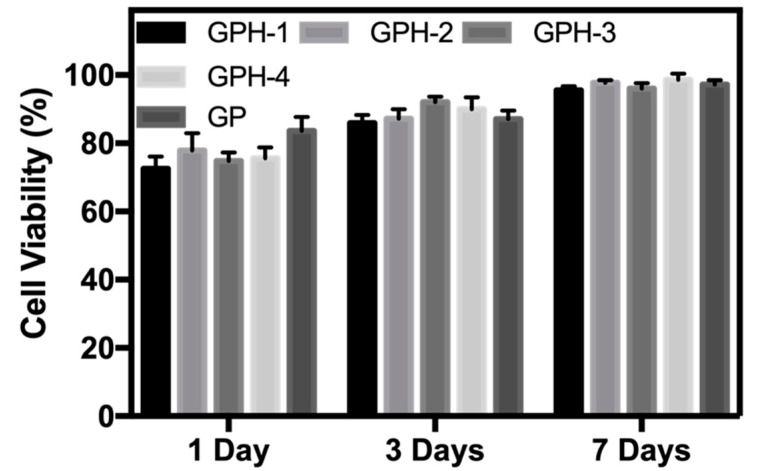
Cell viability of MC3T3-E1 on the surface of composite hydrogel.

After one day of inoculation, MC3T3-E1 adhered well on the surface of the composite hydrogel with five different nHA contents. As can be seen from Figure 9, the MC3T3-E1 cells adhered to the surface of the hydrogel showed a long spindle shape. As the culture time increases, MC3T3-E1 cells can proliferate in all five hydrogel samples, and gradually grow over the surface of the hydrogels. Cell viability was calculated by counting the number of green viable cells and red dead cells in the live/dead staining map of ImageJ, 1, 3, and 7 d, as shown in Figure 10. After one day of inoculation, the cell viability of MC3T3-E1 on the surface of the four GPH composite hydrogel samples was more than 70% and cell viability on the GP hydrogel surface was 83.6% ± 4.1%, which was significantly increased (*p* < 0.05). This phenomenon may be due to the release of some unencapsulated nHA from the hydrogel at the beginning, which affects the cell viability. With the increase of culture time, the cell viability also increased. After three days, the cell viability was greater than 85% and there was no significant difference between the GP and GPH groups. After seven days, cell viability of all the hydrogel samples were greater than 95%. There was no statistically significant difference between the groups at the same culture time. Therefore, compared with the GP group, the four nHA contents used in this experiment had no significant effect on the cell viability of MC3T3-E1. GelMA-PEGDA-nHA composite hydrogel has good biocompatibility.

## 4. Conclusions

The ideal bone defect repair material needs to have good biocompatibility, biodegradability, excellent mechanical properties, good bone induction, and a three-dimensional porous network structure. The preparation of hard and soft tissue materials with good biocompatibility, mechanical properties, and biological activity was a leading topic in the current international biomaterials research.

In this work, a GelMA-PEGDA-nHA ternary composite hydrogel material was successfully prepared. Compared with the previously reported GelMA-PEGDA material, it has stronger mechanical properties, longer degradation time, lower swelling ratio, and also good biocompatibility. The addition of nHA forms a bridge structure [HO]–Ca^2+^–[OH] inside the GelMA-PEGDA hydrogel, which makes the internal structure of the hydrogel more stable, thereby improving the physical and chemical properties. The mechanical strength of the hydrogel can be adjusted by changing the amount of nHA added. It was reported that nHA can promote mineralization, which makes the hydrogel form a biological connection with the autologous bone [32,33]. The photo-crosslinking properties of GelMA and PEGDA enable GelMA-PEGDA-nHA prepolymerization solution to be injected into the defect site to polymerize in situ to form a hydrogel scaffold, which allows a minimally invasive treatment. Additionally, with the development of 3D printing technology in recent years, in conjunction with tomography technology, more and more researchers use bioprinting to prepare bone repair scaffolds that match the shape of the defect. GelMA-PEGDA-nHA is a promising material for bone tissue repair scaffolds.

## Figures and Tables

**Table 1:** Proportion of different components in the sample prepolymer.

Sample	GelMA %	PEGDA %	nHA %	Irgacure2959 %
GPH-1	10	5	0.1	0.2
GPH-2	10	5	1	0.2
GPH-3	10	5	2	0.2
GPH-4	10	5	5	0.2
